# Magnetic resonance imaging findings of intracranial extraventricular ependymoma: A retrospective multi‐center cohort study of 114 cases

**DOI:** 10.1002/cam4.6279

**Published:** 2023-06-27

**Authors:** Liyan Li, Yan Fu, Yinping Zhang, Yipu Mao, Deyou Huang, Xiaoping Yi, Jing Wang, Zeming Tan, Muliang Jiang, Bihong T. Chen

**Affiliations:** ^1^ Department of Radiology First Affiliated Hospital of Guangxi Medical University Nanning P. R. China; ^2^ Department of Radiology Xiangya Hospital, Central South University Changsha P. R. China; ^3^ Department of Radiology Nanning First People's Hospital Nanning P. R. China; ^4^ Department of Radiology Affiliated Hospital of Youjiang Medical University for Nationalities Baise P. R. China; ^5^ National Engineering Research Center of Personalized Diagnostic and Therapeutic Technology Xiangya Hospital Changsha P. R. China; ^6^ National Clinical Research Center for Geriatric Disorders Xiangya Hospital, Central South University Changsha P. R. China; ^7^ Hunan Key Laboratory of Skin Cancer and Psoriasis, Xiangya Hospital Central South University Changsha P. R. China; ^8^ Hunan Engineering Research Center of Skin Health and Disease Xiangya Hospital, Central South University Changsha P. R. China; ^9^ Department of Dermatology Xiangya Hospital, Central South University Changsha P. R. China; ^10^ Department of Neurology Xiangya Hospital, Central South University Changsha P. R. China; ^11^ Department of Neurosurgery Xiangya Hospital, Central South University Changsha P. R. China; ^12^ Department of Diagnostic Radiology City of Hope National Medical Center Duarte California USA

**Keywords:** glioblastoma multiforme (GBM), intracranial extraventricular ependymoma (IEE), magnetic resonance imaging (MRI), visually AcceSAble Rembrandt images (VASARI)

## Abstract

**Background:**

Intracranial extraventricular ependymoma (IEE) is an ependymoma located in the brain parenchyma outside the ventricles. IEE has overlapping clinical and imaging characteristics with glioblastoma multiforme (GBM) but different treatment strategy and prognosis. Therefore, an accurate preoperative diagnosis is necessary for optimizing therapy for IEE.

**Methods:**

A retrospective multicenter cohort of IEE and GBM was identified. MR imaging characteristics assessed with the Visually Accessible Rembrandt Images (VASARI) feature set and clinicopathological findings were recorded. Independent predictors for IEE were identified using multivariate logistic regression, which was used to construct a diagnostic score for differentiating IEE from GBM.

**Results:**

Compared to GBM, IEE tended to occur in younger patients. Multivariate logistic regression analysis identified seven independent predictors for IEE. Among them, 3 predictors including tumor necrosis rate (F7), age, and tumor‐enhancing margin thickness (F11), demonstrated higher diagnostic performance with an Area Under Curve (AUC) of more than 70% in distinguishing IEE from GBM. The AUC was 0.85, 0.78, and 0.70, with sensitivity of 92.98%, 72.81%, and 96.49%, and specificity of 65.50%, 73.64%, and 43.41%, for F7, age, and F11, respectively.

**Conclusion:**

We identified specific MR imaging features such as tumor necrosis and thickness of enhancing tumor margins that could help to differentiate IEE from GBM. Our study results should be helpful to assist in diagnosis and clinical management of this rare brain tumor.

## 1 INTRODUCTION

Ependymomas are rare neuroepithelial tumors, accounting for only 6.9% of CNS tumors diagnosed annually.^1^ According to the World Health Organization 2021 Classification of central nervous system tumors, ependymomas should be classified according to a combination of histological and molecular features into supratentorial, posterior fossa, spinal cord, myxopapillary ependymomas and subependymomas.^2^, ^3^ Although the majority of ependymomas originates as a neoplastic transformation of cells in the ventricular system, it can occur anywhere and could be located in the brain parenchyma as the intracranial extraventricular ependymoma (IEE).^4^, ^5^ The supratentorial ependymoma can cover the cerebral hemisphere, and the posterior fossa ependymoma includes the cerebellum and the brain stem.^6^ For example, Supratentorial Ependymoma with ZFTA‐Fusion (ST‐ZFTA) occurs mostly near the ventricular system but can also occur outside the ventricle and in the cortex.^5^ IEE accounts for approximately 2%–6% of all intracranial gliomas.^7^ On the other hand, glioblastoma multiforme (GBM) is the most common primary malignant central glioma, accounting for 51% of all CNS gliomas.^8^ However, IEE is prone to disseminate via cerebrospinal fluid spread.^9^ There is a risk of metastasis to the whole brain and spinal cord, which made it necessary to assess the entire neural axis. Preoperative diagnosis of IEE allows for early and comprehensive assessment of central nervous system. Therefore, an accurate preoperative diagnosis is necessary for optimizing therapy for IEE.

It is challenging to preoperatively distinguish IEE from GBM. To date, biopsy remains the only reliable method. However, biopsy still has a high probability of misdiagnosis, due to sampling errors in tumors with high heterogeneity, and the neuropathologist's awareness of the disease. In addition, biopsy may have inherent medical risks such as tumor implantation along the biopsy track, bleeding and infection. Non‐invasive methods such as magnetic resonance (MR) imaging have been evaluated. It has been shown that IEE usually occurs in adolescents and young adults, with an expansive growth pattern, which is different from GBM.^10^ However, these features are not sufficient for an accurate preoperative diagnosis of IEE giving the overlapping features between these two brain tumors.

The VASARI (Visually AcceSAble Rembrandt Images) MR imaging feature set is a scoring system for providing consistent description of brain tumor using a set of defined visual features and controlled vocabulary. This feature set currently comprises of 30 morphologic features, describing the location of the tumor, characteristics of the tumor components, and distinct features such as hemorrhage or pial invasion,^11^ which allows for standardized reports largely independent of raters, institutions, or approaches and is optimal for multicenter studies. The VASARI feature set has been adopted to assess primary brain tumors for prediction of survival^12^ and tumor progression.^13^ This feature set has been used frequently on The Cancer Genome Atlas‐GBM dataset due to the dataset's easy accessibility and abundant information.^14^ Although VASARI has been most commonly used to describe tumor morphology of GBM, this feature set has also been applied to lower grade gliomas. For instance, Hyare et al. used these features to predict isocitrate dehydrogenase 1 (IDH1) mutation status.^15^ Zhou et al. aimed at predicting histological grade and tumor progression as well as mutation status (IDH1 and 1p/19q codeletion).^16^ However, the value of VASARI features in diagnosing IEE remains unclear and more work needs to be done to assess the VASARI approach for differentiating IEE from GBM pre‐operatively.

The relationship between GBM and IEE is complex as both are brain tumors but having divergent histopathology and clinical significance. In addition, IEE has overlapping clinical and imaging characteristics with GBM but has different treatment strategy as well as a better prognosis than GBM. An accurate preoperative diagnosis is prudent for optimizing the treatment of IEE. Here, we retrospectively evaluated the MR‐VASARI features in a multicenter cohort of 114 patients with IEE and 258 patients with GBM. The purpose of this study was to identify the potential preoperative MR imaging predictors for distinguishing IEE from GBM through assessment of their MR‐ VASARI and clinicopathological features.

## 2 MATERIALS AND METHODS

### 2.1 Patients

The present study was approved by the Ethics Committee of the participating hospitals including the First Affiliated Hospital of Guangxi Medical University (IRB#:2022‐KY‐E‐(236)), Nanning First People's Hospital (IRB#:2022–196‐01), and Xiangya Hospital, Central South University (IRB#: 202303034), P.R. China and the informed consent was waived due to the retrospective nature of this study.

**FIGURE 1:**
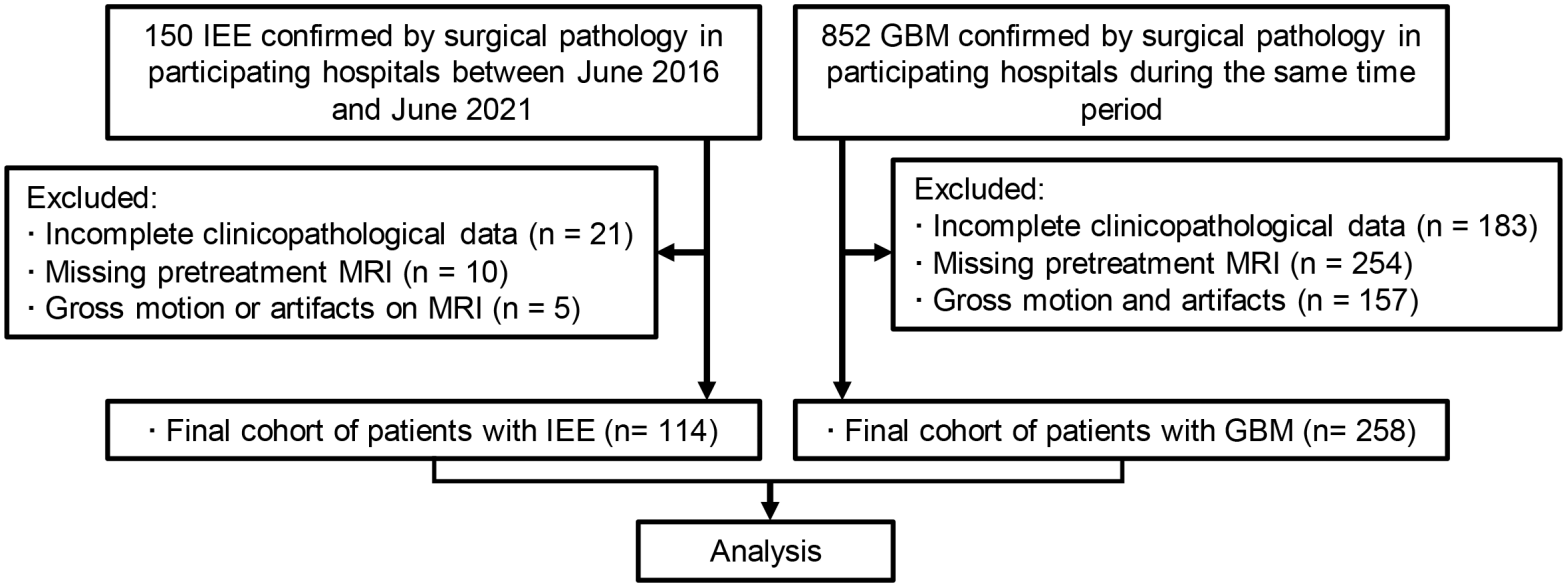
Flowchart for identification of the study cohort consisting of patients with intracranial extraventricular ependymoma (IEE) or glioblastoma (GBM).

Consecutive patients with histopathologically confirmed IEEs and GBMs between June 2016 and June 2021 at the participated hospitals were included in this study. The details of study enrollment and inclusion criteria are presented in Figure 1.

### 2.2 Pathological reassessment

All tumor sections including sections of hematoxylin–eosin staining (H&E staining) and immunohistochemistry (IHC) were collected for reassessment. Paraffin specimens were used for re‐sectioning when needed. Formalin‐fixed paraffin‐embedded sections (4 μm) were stained with hematoxylin–eosin stain and were re‐assessed to confirm the pathology.

All pathological sections in the study cohort were re‐analyzed and diagnosed as IEE and GBM by two neuropathologists (J.Z, and J.H, with 20 and 25 years of experience, respectively). Both neuropathologists were unaware of the clinicopathological data. In case of disagreement when consensus could not be reached between the two primary study neuropathologists, a third experienced neuropathologist (Y.D, with more than 30 years of experience) would make the final diagnosis.

### 2.3 Preoperative MR image acquisition

Brain MR images were obtained in a 3 Tesla GE scanner (GE Medical Systems Discovery MR750w), or a 1.5 Tesla Siemens scanner (Siemens Medical Systems MAGNETOM Avanto or Sempra), or a 3 Tesla Siemens scanner (Siemens Medical Systems Magnetom Verio). Routine standardized brain MR scanning was performed, including T1‐weighted images (T1WI), T1‐weighted with gadolinium contrast‐agent (T1WI + C), T2‐weighted (T2WI), and T2‐weighted with fluid‐attenuated inversion recovery (T2‐FLAIR). All sequences were acquired at the FOV of 220 × 220 mm, slice thickness of 5 mm, matrix of 256 × 256, and slice spacing (1 mm), as indicated in our previous studies.^17^

The MRI images were independently reviewed by two neuroradiologists (X.L and Y.Q, with 15 and 20 years of experience, respectively) who were not aware of the clinicopathological data. Imaging results were recorded by consensus. For the cases that did not reach consensus, a third experienced neuroradiologist (L.L, with more than 30 years of experience) would make the final decision.

### 2.4 MR‐VASARI imaging features

All brain MR images were assessed using the VASARI features. Each lesion was scored according to the VASARI feature set, with 30 imaging features being evaluated (F1‐F30). Among these 30 features, 27 were ultimately used, while three (F26, F27, and F28) were excluded due to the lack of post‐surgical MR images in some patients. A complete description of the VASARI feature set can be found in the reference (https://wiki.nci. nih.gov/display/CIP/VASARI).^18^

### 2.5 Statistical analysis

Statistical analysis was performed with IBM SPSS Statistics (versions 26.0, IBM) and MedCalc Statistics (versions 20.0, IBM). We first performed the Kolmogorov–Smirnov test to assess the normality of all continuous parameters. Mann–Whitney test, *t*‐test, or chi‐square test was applied to compare different types of variables between groups. Univariate and multivariate binary logistic regression was used to identify the features for distinguishing IEE from GBM. Spearman correlation analysis and Pearson correlation analysis were performed to remove potential duplicate factors. Receiver Operating Characteristic curve (ROC) analysis was performed to obtain the area under curve (AUC) values for assessing the prediction efficacy, as well as the threshold values for each feature. A significant difference was defined as *p* < 0.05.

## 3 RESULTS

### 3.1 Clinical data

A total of 114 patients (group 1) with pathologically confirmed IEEs were included in this study, and 258 patients with confirmed GBMs in the same period were included as the group 2 (Figure 1).

There were 78 males and 36 females in Group 1 who had a median age of 22.0 years (IQR: 10.0–44.0 years). There were 164 males and 94 females in Group 2 with a median age of 50.0 years (IQR: 39.0–59.0 years) (Table 1).

**TABLE 1:** Clinicodemographic and MR imaging characteristics assessed with the Visually Accessible Rembrandt Images (VASARI) feature set in the study cohort.

Characteristic	IEE (*n* = 114)	GBM (*n* = 258)	*p*‐value
Demographics and clinical characteristics			
Gender, *n* (%)			0.365
Male	78 (68.4%)	164 (63.6%)	
Female	36 (31.6%)	94 (36.4%)	
Age (median [IQR], years)	22.0 (10.0–44.0)	50.0 (39.0–59.0)	<0.001
Laboratory findings (mean, [SD])			
PLT	277.44 (110.11)	211.4 (75.66)	<0.001
RDW	9.89 (5.96)	13.10 (1.54)	<0.001
HGB	124.97 (19.30)	133.13 (17.47)	<0.001
MR‐VASARI features (mean, [SD])			
F1	3.04 (2.49)	3.29 (2.71)	<0.001
F3	1.64 (1.16)	2.22 (1.21)	<0.001
F7	3.42 (0.83)	4.87 (1.12)	<0.001
F10	1.61 (0.56)	1.36 (0.58)	<0.001
F11	3.96 (0.24)	3.34 (0.85)	<0.001
F16	1.30 (0.46)	1.46 (0.50)	<0.001
F24	1.03 (0.16)	1.32 (0.47)	<0.001
F30	13.04 (4.26)	10.85 (3.37)	<0.001

*Note*: MR‐VASARI features: F1, tumor locations; F3, eloquent brain; F7, proportion necrosis; F10, T1/FLAIR ratio; F11, thickness of enhancing margin; F16, tumor hemorrhage; F24, satellites of tumor; F30, tumor size (the largest perpendicular (x‐y) cross‐sectional diameter of T2 signal abnormality).

Abbreviations: GBM, glioblastoma multiforme, HGB, hemoglobin; IEE, intracranial extraventricular ependymoma; PLT, platelet count; RDW, red blood cell distribution width.

When compared to patients with GBM, patients with IEE tended to be at a younger age of onset (median age of 22.0 years for IEE vs. median age of 50.0 years for GBM), having a decreased serum level of red blood cell volume distribution width (RDW) (9.89 ± 5.96 vs. 13.10 ± 1.54). There were significant differences in blood testing results between Group 1 and Group 2 (*p* < 0.05) (Table 1).

### 3.2 MR‐VASARI features

The inter‐observer intraclass correlation (ICC) values of MR‐VASARI features extracted by reader 1 and reader 2 in their first extraction ranged from 0.756 to 0.992. The intra‐observer ICC values for two extractions for each case performed by reader 1 ranged from 0.740 to 0.986.

**FIGURE 2:**
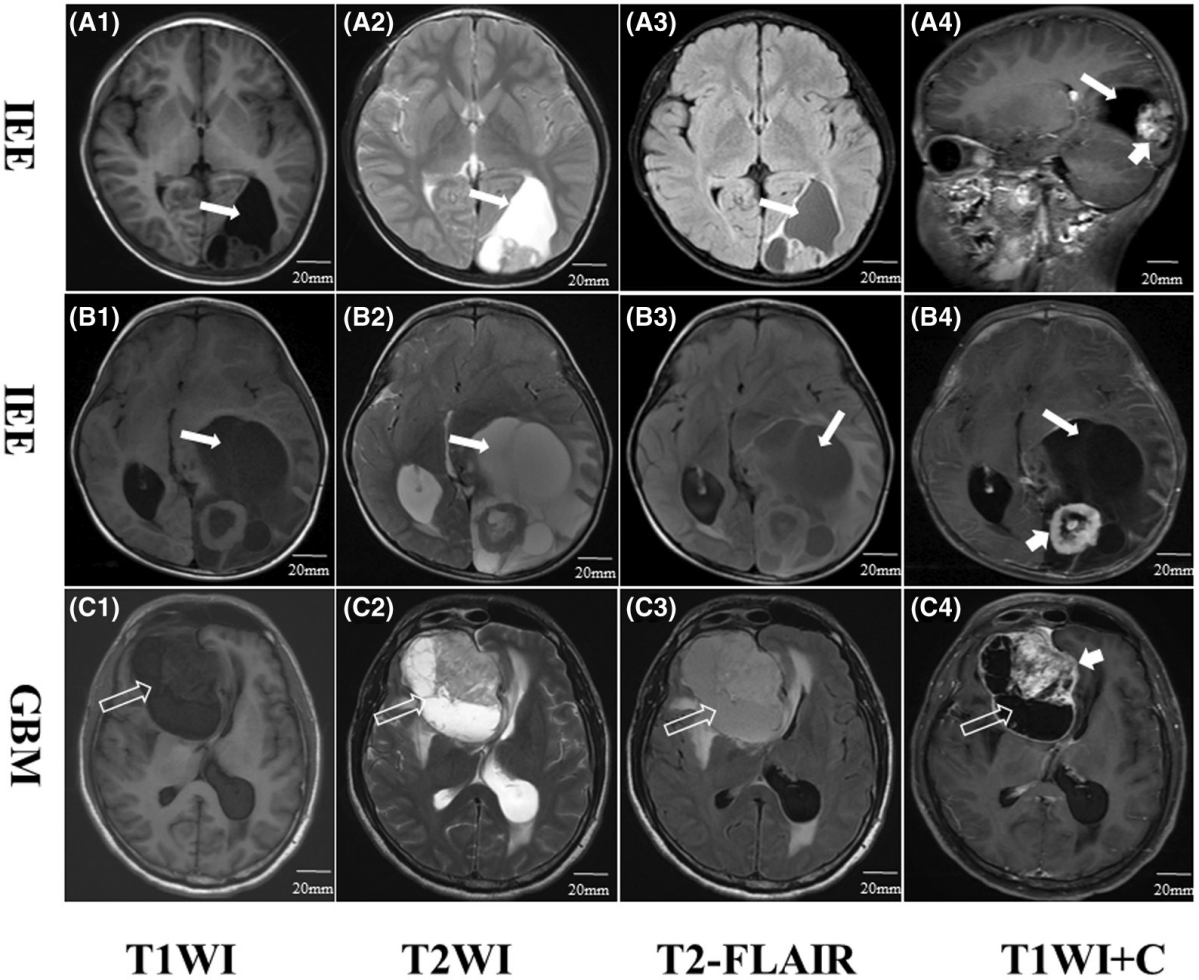
Representative MR images of intracranial extraventricular ependymoma (IEE) and glioblastoma multiforme (GBM). Figures A1–A4: A 3‐year‐old female with IEE (Case 95). Figures B1–B4: A 35‐year‐old female with IEE (Case 100). Figures C1–C4: A 57‐year‐old male with GBM (Case 77). The hollow arrowheads represent tumor necrosis (Figure C1–C4), the long arrows indicate the cystic area within the tumor (Figure A,B). The short arrows represent peripheral enhancing margin (Figure A–C).

Figure 2 presents the characteristic MR imaging findings of IEE. In contrast to GBM, IEE was mostly located in non‐functional brain area, with a larger diameter in axial T2WI, less tumor necrosis or hemorrhage, and less likely to have satellite foci. On MR images, IEE usually has a mixed cystic and solid appearance, the tumor parenchyma showed slightly iso‐to‐low intensity on T1WI, slightly moderate‐to‐high intensity on T2WI, and moderate‐to‐high intensity on T2‐FLAIR, with obvious cystic degeneration and necrosis (Figure 2). After administration of contrast agent, the tumor showed significant enhancement in the solid component, but no enhancement in the cystic, necrotic or calcified regions (Figure 2) (Figure S1).

**FIGURE 3:**
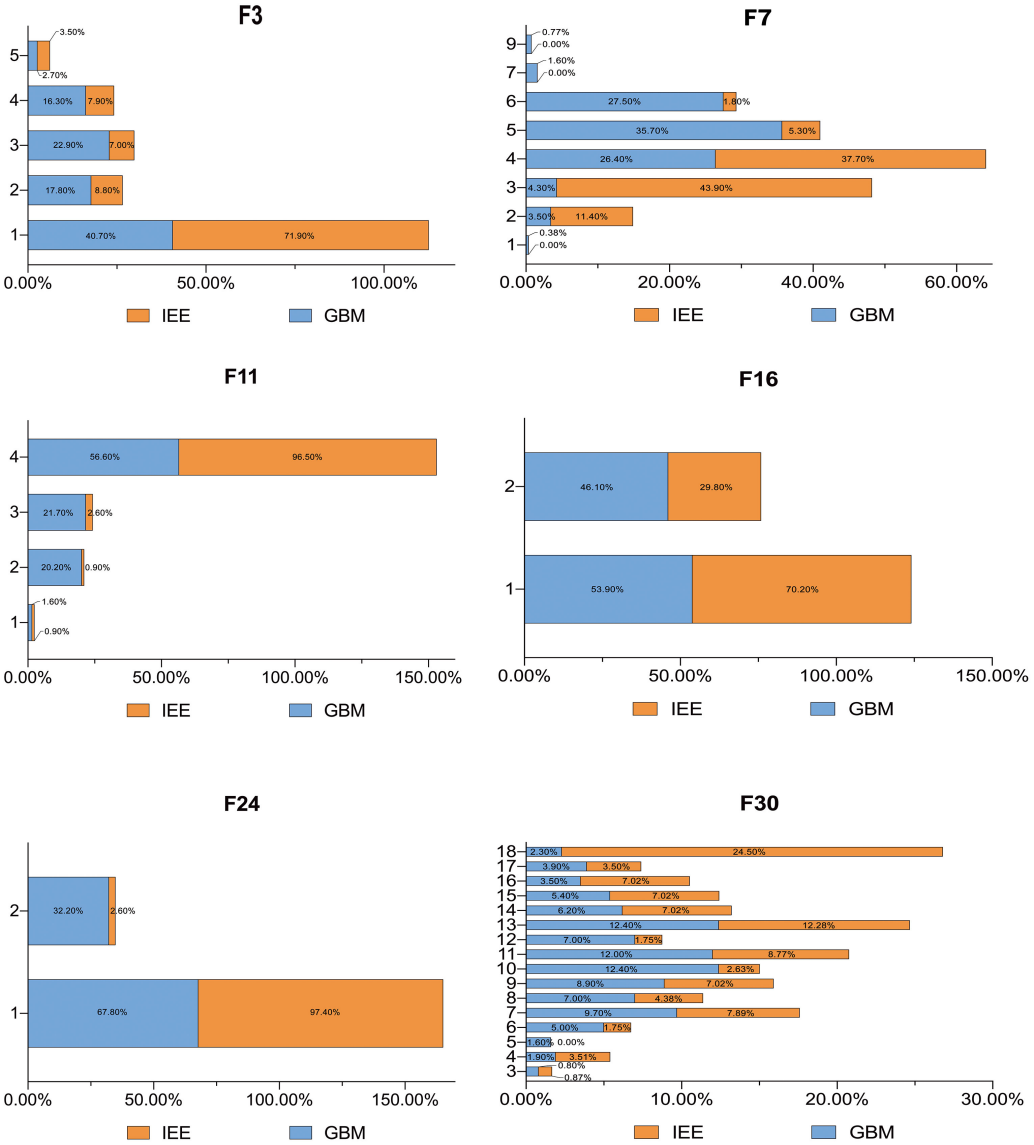
Distribution of the MR‐Visually Accessible Rembrandt Images (VASARI) features in patients with intracranial extraventricular ependymoma (IEE) and glioblastoma multiforme (GBM). Each proportion corresponds to the ratio: number of lesions with the feature / total number of lesions. The orange color represents IEE and the blue color represents GBM. F3, eloquent brain (1 = None; 2 = Speech motor; 3 = Speech receptive; 4 = Motor; 5 = Vision). F7, proportion necrosis (1 = n/a; 2 = None (0%); 3 = <5%; 4 = 6–33%; 5 = 34–67%; 6 = 68–95%; 7= > 95%; 8 = 100%; 9 = Indeterminate). F11, the thickness of enhancing margin (1 = n/a; 2 = None; 3 = Thin; 4 = Thick/solid). F16, hemorrhage of the tumor (1 = No; 2 = Yes). F24, indicating the satellites of tumor (1 = No; 2 = Yes). F30, tumor size: the largest perpendicular (x‐y) cross‐sectional diameter of T2 signal abnormality. (1 = <0.5 cm; 2 = 0.5 cm; 3 = 1.0 cm; 4 = 1.5 cm; 5 = 2.0 cm; 6 = 2.5 cm; 7 = 3.0 cm; 8 = 3.5 cm; 9 = 4.0 cm;10 = 4.5 cm; 11 = 5.0 cm; 12 = 5.5 cm; 13 = 6.0 cm;14 = 6.5 cm; 15 = 7.0 cm; 16 = 7.5 cm; 17 = 8.0 cm; 18= > 8.0 cm.)

The differences in MR‐VASARI image features between IEE and GBM are presented in Figure 3. When compared to GBM, IEE usually appeared as larger mass lesions (F30, 13.04 ± 4.26) with thicker enhancing margins (F11, 3.96 ± 0.24), but less likely to have satellite foci (F24, *n* = 3, 2.60%), to involve eloquent functional brain regions (F3, *n* = 32, 28.1%), to have hemorrhage (F16, *n* = 34, 29.8%), or to have intra‐tumoral necrosis (F7, *n* = 51, 44.7%) (Table 1).

### 3.3 Pathologic findings

**FIGURE 4:**
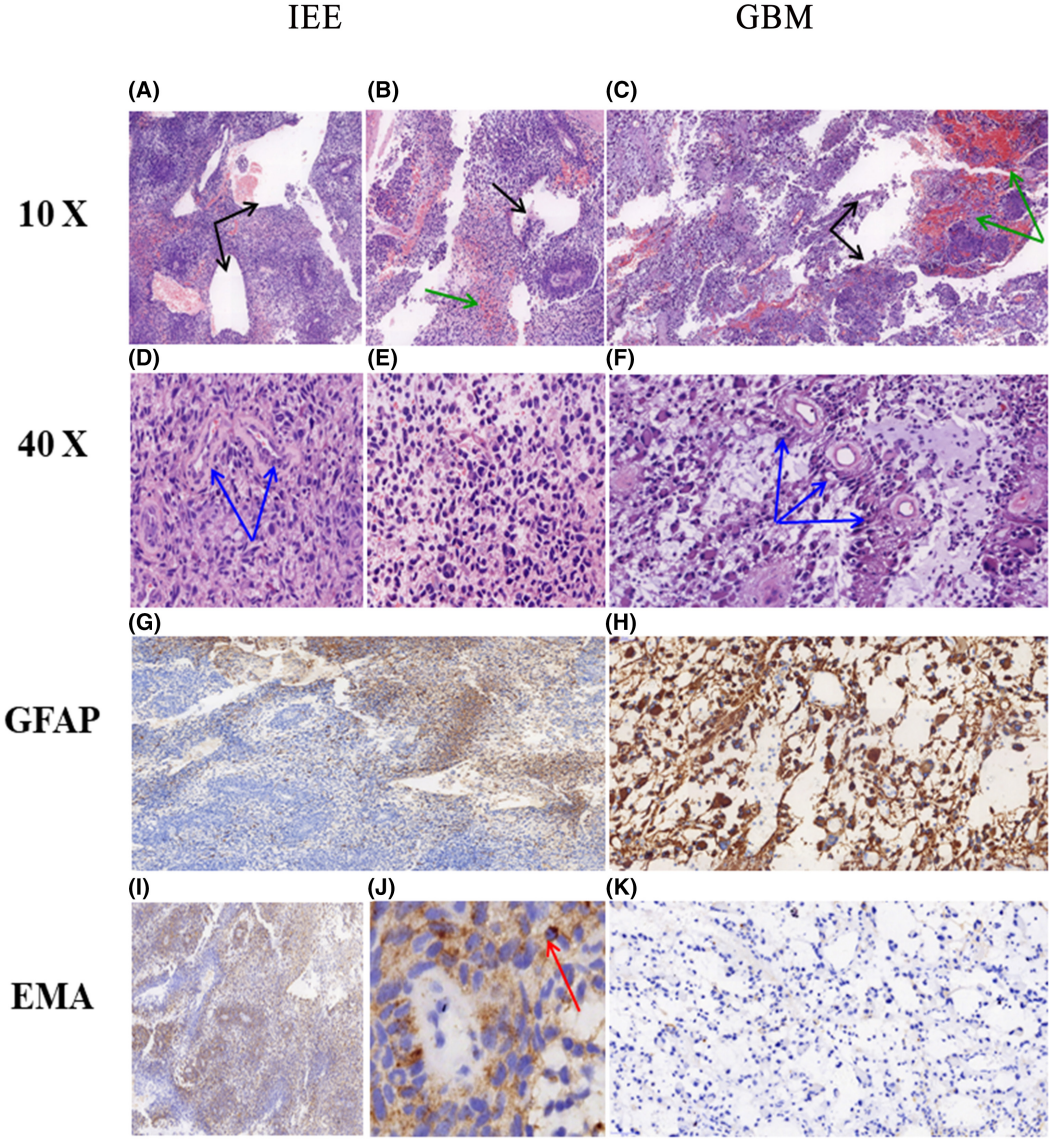
Pathological features of intracranial extraventricular ependymoma (IEE) and glioblastoma multiforme (GBM). H & E staining shows hemorrhage (green arrows, B, C), cystic degeneration (black arrows, A, B, C) and abnormally proliferated blood vessels (blue arrows, D, F). The immunohistochemistry (IHC) shows positive immunostaining for glial fibrillary acid protein (GFAP) expression in IEE tumor cells with perivascular pseudorosettes (I) and characteristic perinuclear dot‐like labelling in the immunostaining for epithelial membrane antigen (EMA) (red arrows, J). GBM tumor cells are positive for GFAP but do not express EMA. (Original magnification, 10× and 40×).

Formalin‐fixed paraffin‐embedded sections (4 μm) stained with H&E and IHC stain were comprehensively analyzed. The characteristic pathological findings of IEE were the following: a daisy‐shaped mass of the ependyma, that is, the middle part consisted of empty blood vessels, and the surrounding area consisted of powdered unstructured area, followed by garland‐like structure. As showed in Figure 4I,J, the surrounding parenchyma had a clear boundary showing the perivascular pseudorosettes, and characteristic perinuclear dot‐like labelling on the immunostaining images for epithelial membrane antigen (EMA). Immunostaining for glial fibrillary acidic protein (GFAP) showed widespread labelling in the cytoplasm and processes of the tumor cells (Figure 4) in IEE. In addition, there were scattered cystic areas (Figure 4A–C) and hemorrhage (Figure 4B,C) identified on H&E staining images for IEE.

The presence of cystic areas and hemorrhage in IEE was obviously less than GBM, which was consistent with the MR image findings. Of note, GBM had more abnormally proliferating vascular masses than IEE, which might be partly due to the rapidly growing tumor cells leading to vascular proliferation and abnormal vascular system in GBM.^19^

### 3.4 Predictors for IEE

On univariate logistic regression analysis, 13 features for distinguishing IEE from GBM were identified (Table 2). To avoid the potential risk for over fitting since there were 114 patients with IEE, the number of features included in the multivariate logistic regression should not exceed 11. The result of correlation analysis showed a correlation coefficient of 0.44 for platelet account and age, and the platelet account was removed prior to multivariate logistic regression analysis (Table 2). Subsequently, based on these remaining 11 features, multivariate logistic regression analysis identified 8 independent predictors including age, red blood cell volume distribution width (RDW), functional brain area involvement (F3), tumor necrosis ratio (F7), thickness of tumor enhancement margin (F11), presence of tumor hemorrhage (F16), satellite foci (F24), and the largest perpendicular (x‐y) cross‐sectional diameter on T2WI (F30).

**TABLE 2:** Predictors identified through univariate analysis for subsequent multivariate analysis.

Characteristic	Univariate analysis		Multivariate analysis	
	OR (95% CI)	*p*‐value	OR (95% CI)	*p*‐value
Age	0.95 (0.93–0.96)	<0.001	0.936 (0.904–0.968)	<0.001
PLT	1.01 (1.00–1.01)	<0.001	–^a^	–^a^
RDW	0.79 (0.72–0.86)	<0.001	0.76 (0.63–0.91)	0.003
MPV	0.68 (0.59–0.79)	<0.001	0.93 (0.68–1.28)	0.664
BUN	0.54 (0.45–0.64)	<0.001	0.76 (0.54–1.06)	0.105
F1	0.97 (0.89–1.05)	0.442	–	–
F3	0.65 (0.52–0.80)	<0.001	0.43 (0.26–0.71)	0.001
F7	0.26 (0.19–0.35)	<0.001	0.17 (0.09–0.31)	<0.001
F10	2.02 (1.40–2.92)	<0.001	2.10 (0.86–5.13)	0.103
F11	9.81 (4.15–23.16)	<0.001	27.59 (6.78–112.32)	<0.001
F16	0.50 (0.31–0.79)	0.003	0.18 (0.06–0.62)	0.006
F24	0.06 (0.02–0.19)	<0.001	0.02 (0.00–0.17)	<0.001
F30	1.17 (1.10–1.25)	<0.001	1.43 (1.23–1.66)	<0.001

*Note*: MR‐VASARI (Visually Accessible Rembrandt Images) features: F1, tumor locations; F3, eloquent brain; F7, proportion necrosis; F10, T1/FLAIR ratio; F11, thickness of enhancing margin; F16, tumor hemorrhage; F24, satellites of tumor; F30, tumor size (the largest perpendicular (x‐y) cross‐sectional diameter of T2 signal abnormality).

Abbreviations: PLT, Platelet count; RDW, Red blood cell volume distribution width; MPV, mean platelet volume; BUN, blood urea nitrogen; OR, odds ratio.

^a^ PLT was excluded from the multivariate logistic regression analysis due to age and PLT correlation coefficient was too high.

**FIGURE 5:**
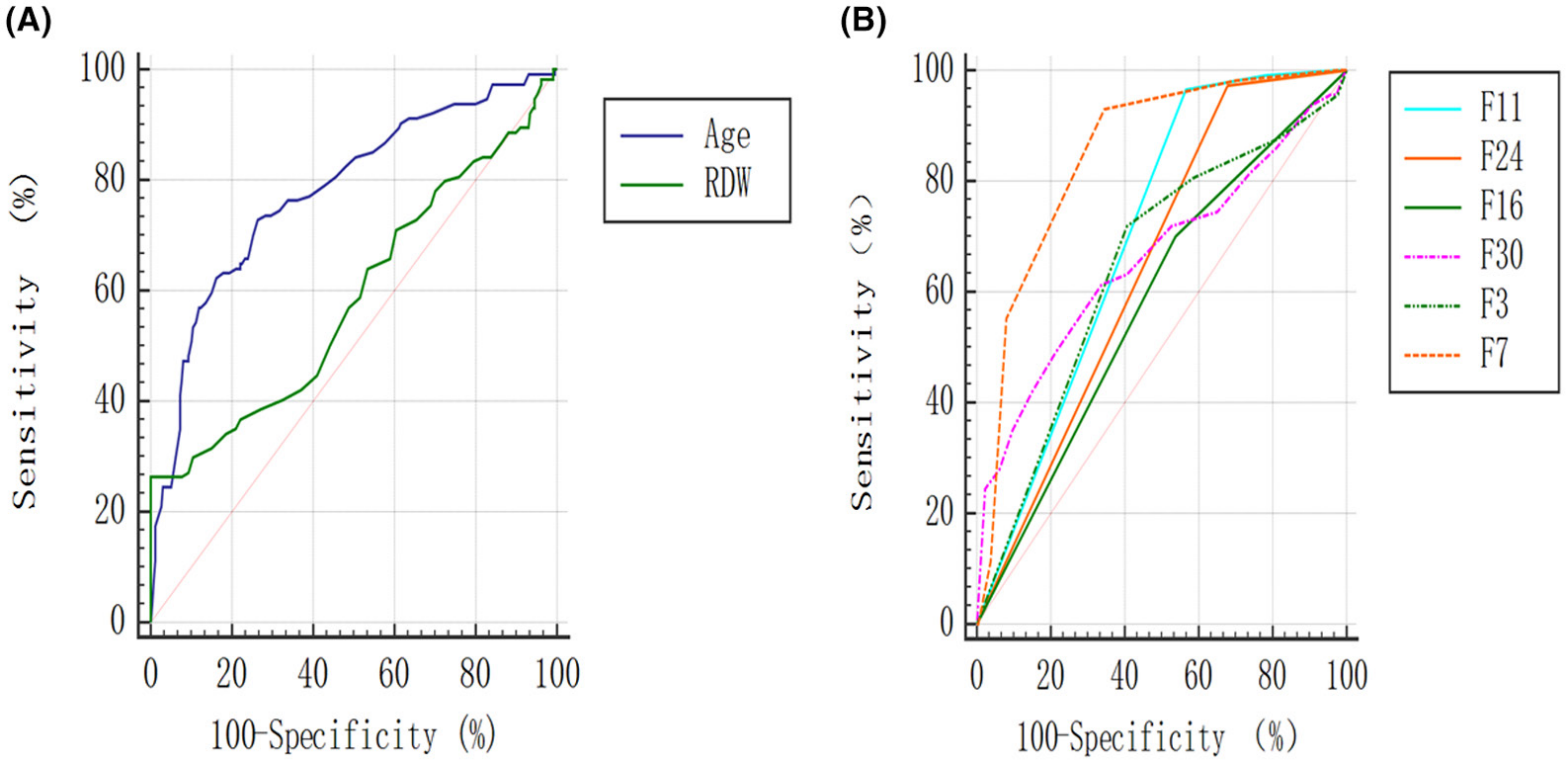
Receiver operating characteristic (ROC) curve analysis for identification of predictors to differentiate intracranial extraventricular ependymoma (IEE) from glioblastoma (GBM). (A) ROC curve analysis for age (solid purple line) and red blood cell volume distribution width (RDW) (solid green line). (B) ROC curves for the MR‐Visually Accessible Rembrandt Images (VASARI) features. F3, eloquent brain; F7, proportion necrosis; F11, thickness of enhancing margin; F16, hemorrhage of the tumor; F24, satellites of tumor; F30, tumor size (the largest perpendicular (x‐y) cross‐sectional diameter of T2 signal abnormality).

Finally, 3 predictors including age, F7 and F11 with high performance for distinguishing IEE from GBM were extracted from the 8 predictors from multivariate analysis through ROC analysis (Table 3, Figure 5). The AUC for F7, age and F11 were 0.85 (95% confidence interval [95% CI]: 0.81–0.88), 0.78 (95% CI: 0.73–0.82), 0.70 (95% CI: 0.65–0.75) respectively; with a sensitivity of 92.98% (95% CI: 90.38%–95.58%), 72.81% (95% CI: 68.29%–77.33%), 96.49% (95% CI: 94.62%–98.36%), and a specificity of 65.50% (95% CI: 60.67%–70.33%), 73.64% (95% CI: 69.12%–78.12%), 43.41% (95% CI: 38.37%–48.45%).

**TABLE 3:** Predictors for differentiating intracranial extraventricular ependymoma (IEE) from glioblastoma multiforme (GBM) through receiver operating characteristic curve (ROC) analysis.

Tumor characteristic	Cutoff value	Sensitivity (%)	Specificity (%)	Youden Index	AUC (95% CI)
Age	40	72.81	73.64	0.46	0.78 (0.73–0.82)
RDW	0.16	26.32	100	0.26	0.59 (0.53–0.64)
F3	1	71.93	59.3	0.31	0.65 (0.60–0.70)
F7	4	92.98	65.50	0.58	0.85 (0.81–0.88)
F11	3	96.49	43.41	0.40	0.70 (0.65–0.75)
F16	1	70.18	46.12	0.16	0.58 (0.53–0.63)
F24	1	97.37	32.17	0.30	0.65 (0.60–0.70)
F30	13	78.68	49.12	0.28	0.66 (0.61–0.71)

*Note*: F3, eloquent brain; F7, proportion necrosis; F11, thickness of enhancing margin; F16, tumor hemorrhage; F24, satellites of tumor; F30 tumor size (the largest perpendicular (x‐y) cross‐sectional diameter of T2 signal abnormality).

Abbreviations: AUC, area under the curve; RDW, red blood cell volume distribution width.

## 4 DISCUSSION

In this study, we identified specific MR imaging features and clinical characteristics that were more commonly seen in IEE than GBM. To the best of our knowledge, this was the first study to comprehensively analyze IEE tumor morphology in the largest cohort on this rare malignant brain tumor in comparison to GBM using the MR‐VASARI features.

Brain MRI is an essential tool for diagnosis and treatment response evaluation of GBM and IEE, which is intertwined in all aspects of clinical management of patients with brain tumor. Advanced MRI sequences include diffusion‐weighted imaging and perfusion‐weighted imaging could improve the ability to distinguish between ependymoma and medulloblastoma^20^ and assess the growth characteristics of high‐grade gliomas such as GBM.^21^ Our study showed that there was a lower proportion of necrosis in IEE when compared with GBM, which might be due to the different growth patterns of these tumors. Tumor necrosis occurs when the rapidly enlarging tumor exceeding its nutritional supply for metabolism.^22^ IEE tended to have an expansive growth pattern with a relatively slower growth rate than GBM,^10^ which may accommodate the growth of tumor cells better than GBM and therefore was less prone to necrosis. In addition, it has been reported the telomere length of cancer cells is related to cell senescence and necrosis.^23^ Typical ependymal cells are mitotically inactive and the telomere length of cancer cells remain stable, leading to less occurrence of senescence and necrosis.^24^ Furthermore, telomere homeostasis is regulated by telomerase, an RNA‐dependent DNA polymerase^25^ whose activity is highly upregulated in 90% of malignant tumors.^26^, ^27^ Since GBM is highly malignant, it seems reasonable to assume that there would be more upregulation of telomerase in GBM than IEE, thus leading to more necrosis in GBM.

Our results showed a lower possibility of hemorrhage in IEE than GBM. Studies have shown that invasive growth of malignant tumor cells can disrupt vascular integrity by invading the perivascular space and vessel wall.^28^ Notably, GBM increases procoagulant factors such as tissue factor (TF) and plasminogen activator inhibitor‐1 (PAI‐1) and decreases plasminogen activation level of fibrin clot (tPA) causing fibrin clot formation, resulting in vascular lumen occlusion and increased hemorrhage.^29^ On the other hand, IEE does not produce procoagulant factors such as TF and PAI‐1, and does not cause intravascular thrombosis and blood flow impairment, and is therefore less likely to bleed as compared to GBM.

We observed that IEE tended to present a thicker tumor‐enhancing margin than GBM, which could be explained by vascular permeability. Tumor enhancement is related to the destruction of the blood–brain barrier (BBB) and the increase of vascular permeability, resulting in increased uptake of contrast agents by tumor cells.^30^ Studies have found that the BBB is dysplastic in astrocytoma, oligodendroglioma, and ependymoma, leading to increased vascular permeability.^31^ Moreover, ependymomas are slow‐growing tumors with an abnormal vascular system,^32^, ^33^ which can overexpress vascular endothelial growth factor (VEGF), a major marker of vascular growth.^34^, ^35^ Thus, the increased microvasculature of ependymoma and the increased vascular permeability lead to more tumor enhancement. On the contrary, as a highly heterogeneous malignant tumor with aggressive growth,^30^, ^36^ GBM tumor cells migrate further outside the normal brain parenchyma behind the intact blood–brain barrier and therefore showing less enhancement.^30^, ^37^ In addition, GBM tends to show thinner enhancing margins with more tumor necrosis as compared to IEE.

Our study also found that a younger age of onset for IEE with a median age of 22 years, compared with 50 years for GBM. This was consistent with previous findings that ependymoma is a rare primary central nervous system tumor that commonly occurs in children and adolescents, whereas GBM occurs mostly in adults.^38^, ^39^

IEE should also be differentiated from other brain tumors such as brain metastases, anaplastic astrocytoma, oligodendrogliomas, etc. Brain metastases were the most common tumors of the central nervous system, often presenting as small nodules with large amount of edema.^40^ Brain metastases were spread by hematogenous route while IEE may spread through cerebrospinal fluid. Anaplastic astrocytoma are usually not accompanied by cortical involvement, with homogeneous signal intensity on T2‐weighted images and often shows nodular enhancement on contrast‐enhanced scans.^41^ When calcification is noted in ependymoma, it should be differentiated from oligodendrogliomas which are mostly non‐enhancing.^42^

There are some limitations to this study. First, our study was retrospective, covering a long time span, and some pathological specimens were missing, which prevents us from performing molecular typing analysis. Second, considering the retrospective nature of this study, selective bias was unavoidable. Some patients with more advanced brain tumors were referred from community hospitals for higher level care with more specialized neuro‐oncology services in the participating hospitals. These patients' clinical and image data was not stored permanently in our medical records and were not available for the precise imaging‐pathological correlation. Third, our sample size was still modest due to the rarity of IEE and we did not have sufficient statistical power for additional analysis such as assessing the imaging features of IEE according to their pathological grades. Fourth, the imaging features identified in our study all belonged to the visual image features evaluated by neuroradiologists, with lack of objective quantitative criteria for parameters such as the degree of thickening of the tumor margin. In addition, assessment of the MR‐VASARI imaging features may partly depend on the neuroradiologists' experience. Lastly, we included only MR images in this study. Incorporation of other imaging modality such as CT, positron emission tomography (PET) CT and PET MRI may help to identify hemorrhage, calcifications and metabolic information of the brain tumors.

## 5 CONCLUSION

Our multicenter study was the first attempt to use the neuroimaging features from the MR‐VASARI feature set for distinguishing IEE from GBM. We observed that IEE had a low proportion of necrosis, a low likelihood of hemorrhage, a thick tumor‐enhanced margins and a younger onset age than GBM. In addition, our study also provided the initial evidence for MR imaging‐based predictive modeling as a non‐invasive approach to diagnose the IEE prior to surgery. Our study results should advance our understanding of diagnosis and treatment of brain tumors.

## AUTHOR CONTRIBUTIONS

**Liyan Li:** Writing – original draft (lead). **Yan Fu:** Writing – original draft (equal). **Yinping Zhang:** Writing – original draft (equal). **Yipu Mao:** Resources (equal). **Deyou Huang:** Resources (equal). **xiaoping yi:** Writing – review and editing (equal). **Jing Wang:** Resources (equal). **Zeming Tan:** Resources (equal); writing – review and editing (equal). **Muliang Jiang:** Resources (equal); writing – review and editing (equal). **Bihong T. Chen:** Writing – review and editing (equal).

## FUNDING INFORMATION

This research was funded by Xiangya‐Peking University, Wei Ming Clinical and Rehabilitation Research Fund (No. xywm2015I35), the Project Program of National Clinical Research Center for Geriatric Disorders (Xiangya Hospital, Grant No. 2022LNJJ09), Natural Science Foundation of Hunan Province (2022JJ30979), China Post‐Doctoral Science Foundation (2018 M632997, 2022 M713536), the Natural Science Foundation of Guangxi (Grant No. 2020GXNSFAA259047), the Clinical Research “Climbing” Program of the First Affiliated Hospital of Guangxi Medical University (Grant No. YYZS2020021), the Youth Science Foundation of the First Affiliated Hospital of Guangxi Medical University (GXMUYSF202216), and the “Medical Excellence Award” Funded by the Creative Research Development Grant from the First Affiliated Hospital of Guangxi Medical University.

## ETHICS STATEMENT

This study was approved by the Ethics Committee of the First Affiliated Hospital of Guangxi Medical University (IRB#:2022‐KY‐E‐(236)), Nanning First People's Hospital (IRB#:2022–196‐01), and Xiangya Hospital, Central South University (IRB#: 202303034), P.R. China and the informed consent was waived due to the retrospective nature of this study.

## Supporting information


Figure S1.

## Data Availability

The data underlying this article will be shared on reasonable request to the corresponding author.
