# Supplementary material for: Magnetic resonance imaging findings of intracranial extraventricular ependymoma: A retrospective multi‐center cohort study of 114 cases
Source: Cancer Med. 2023 Jun 27;12(15):16195–206. doi: 10.1002/cam4.6279 (PMC10469843; doi:10.1002/cam4.6279)
Supplement: Supplementary file 1 — Figure S1. [file CAM4-12-16195-s001.docx]

**Supplementary** Representative MRI images of the intracranial extraventricular ependymoma (IEE).


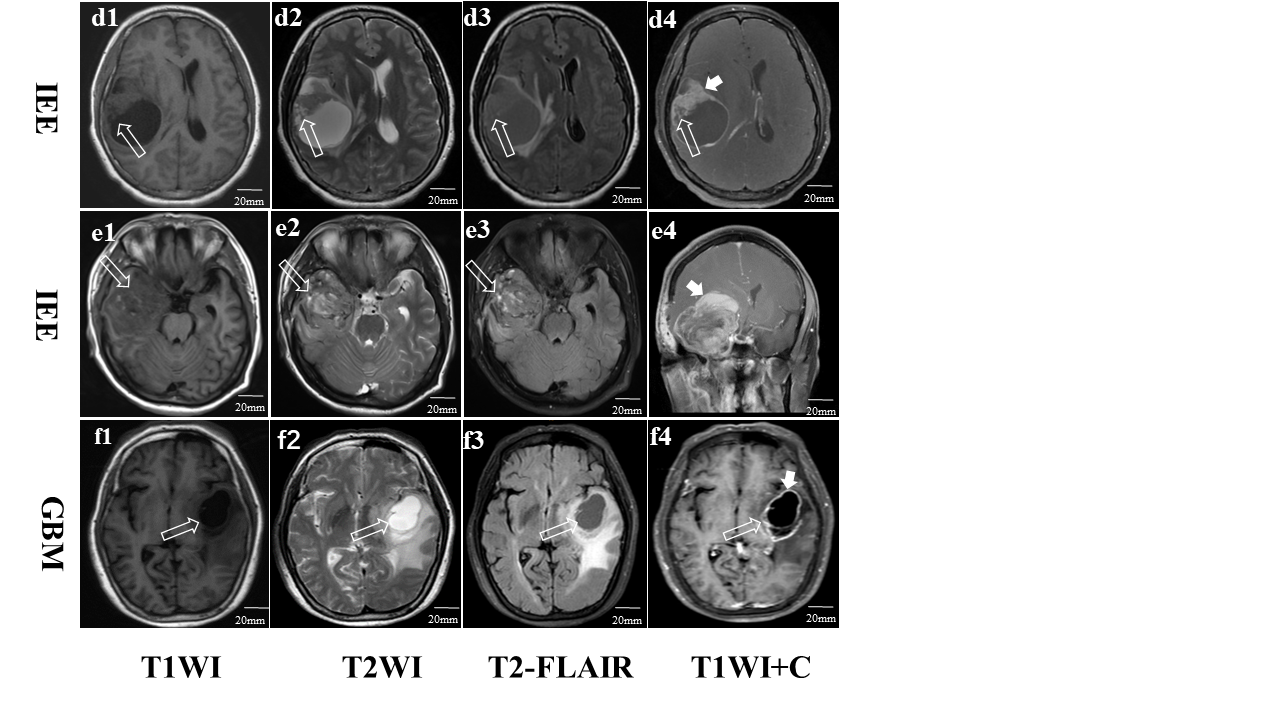


Note：Case 75 (IEE, d1-4), a 50 years’ male. Case 45 (IEE, e1-4), a 5 years’ male. Case 23 (GBM, f1-4), a 61 years’ female. The hollow arrowheads represent necrosis areas of the tumor that T1WI hypointense signal, T2WI hyperintense signal, T2-FLAIR equal or hyperintense signal, and T1C shows nonenhance and low signal with irregular shape. The short arrows represent edge enhanced thickness, which is hyperintense on T1C. In case 75 and 45（d4, e4）, the tumor with thick or nodular enhancing margins. In case 23 (f4), the tumor had more necrosis and the enhancement margin was thin.
